# Numerical modeling and verification of a sonobioreactor and its application on two model microorganisms

**DOI:** 10.1371/journal.pone.0229738

**Published:** 2020-03-11

**Authors:** Nasim Najjarzadeh, Adolf Krige, Taraka R. K. Pamidi, Örjan Johansson, Josefine Enman, Leonidas Matsakas, Ulrika Rova, Paul Christakopoulos

**Affiliations:** 1 Division of Chemical Engineering, Biochemical Process Engineering, Department of Civil, Environmental and Natural Resources Engineering, Luleå University of Technology, Luleå, Sweden; 2 Division of Operation, Engineering Acoustics, Maintenance and Acoustics, Department of Civil, Environmental and Natural Resources Engineering, Luleå University of Technology, Luleå, Sweden; Tallinn University of Technology, ESTONIA

## Abstract

Ultrasound has many uses, such as in medical imaging, monitoring of crystallization, characterization of emulsions and suspensions, and disruption of cell membranes in the food industry. It can also affect microbial cells by promoting or slowing their growth and increasing the production of some metabolites. However, the exact mechanism explaining the effect of ultrasound has not been identified yet. Most equipment employed to study the effect of ultrasound on microorganisms has been designed for other applications and then only slightly modified. This results in limited control over ultrasound frequency and input power, or pressure distribution in the reactor. The present study aimed to obtain a well-defined reactor by simulating the pressure distribution of a sonobioreactor. Specifically, we optimized a sonotrode to match the bottle frequency and compared it to measured results to verify the accuracy of the simulation. The measured pressure distribution spectrum presented the same overall trend as the simulated spectrum. However, the peaks were much less intense, likely due to non-linear events such as the collapse of cavitation bubbles. To test the application of the sonobioreactor in biological systems, two biotechnologically interesting microorganisms were assessed: an electroactive bacterium, *Geobacter sulfurreducens*, and a lignocellulose-degrading fungus, *Fusarium oxysporum*. Sonication resulted in increased malate production by *G*. *sulfurreducens*, but no major effect on growth. In comparison, morphology and growth of *F*. *oxysporum* were more sensitive to ultrasound intensity. Despite considerable morphological changes at 4 W input power, the growth rate was not adversely affected; however, at 12 W, growth was nearly halted. The above findings indicate that the novel sonobioreactor provides an effective tool for studying the impact of ultrasound on microorganisms.

## Introduction

Ultrasound, which comprises sound at frequencies above 20 kHz, has many uses in various industries. For example, high-frequency (1–10 MHz) low-energy diagnostic ultrasound is routinely used for ultrasound imaging in the medical field to evaluate the state of internal tissue structures without changing the physicochemical properties of the tissue [1]. Low-power ultrasound with frequencies of 0.1–1 MHz is used extensively in several food science applications, such as the monitoring of crystallization or the characterization of emulsions and suspensions [2]. The low-power levels required by these applications do not induce changes in the material. On the opposite end, high-intensity ultrasound at lower frequencies (20–100 kHz) can be used as an invasive technique to purposely alter the properties of materials. High-power (10–10 000 W/cm^2^) acoustic waves induce the formation of cavities as they travel through a medium, resulting in physicochemical changes to the material [1]. For example, ultrasound is commonly used to disrupt cells and release intracellular products [3]. In one study, ultrasound was successfully used in lab scale to prevent algal blooms in the lakes or rivers with the removal rate of 86.4% [4]. Intense ultrasound is even known to damage macromolecules such as enzymes [5]. Moreover, ultrasound was successfully used as an eco-friendly pretreatment method of lignocellulosic and waste material [6–7].

Microbial cells can vary substantially with regard to how they are affected by continuous or intermittent ultrasonication. For example, the cyanobacterium *Anabaena flos-aquae* exhibited enhanced growth rate and a 46% increase in biomass yield when intermittently sonicated (5 min/day, 20 kHz, 50 W pulse) [8,9]. In contrast, the same treatment slowed the growth of the microalgae *Selenastrum capricornutum* [10]. Stimulation of *Panax ginseng* suspended plant cells with low doses of ultrasound (0.5–6.0 min, 38.5 kHz, power ≤100 kW/m^3^) induced secondary metabolite production and affected cross-membrane ion fluxes within 2 min of exposure [11]. Based on the research, we can also use ultrasound to improve the fermentation yield [12]. Studies have also shown enhanced rates of enzyme-catalyzed reactions when using ultrasonication [13]. Improved mass transfer (including increased permeability of the cell membrane toward nutrients, oxygen, and cell products) and cell retention (for high-density cultures) have often been cited as possible mechanisms responsible for the ultrasonic enhancement of a culture’s metabolism. However, the exact mechanism has not been clearly identified and more work is required in this sense [10].

To study the effect of sound waves on microbial activity, different types of ultrasound equipment have been developed over the past few decades. However, such equipment is usually obtained by simply combining a standard ultrasound device (such as an ultrasound horn or an ultrasound bath) with common bacterial growth reactors [14–15]. One of the most common methods is to submerge a flask or bottle into the liquid of an ultrasonic cleaning bath. This strategy relies heavily on the functionality of the bath itself and is affected by factors, such as uniformity of sound distribution, placement of the sonotrodes, and temperature. Furthermore, ultrasound intensity itself is strongly dependent on the location of the flask in the bath [16,17]. This could impact the repeatability of experiments if the flasks are not placed in the exact same location. Finally, the frequency and sometimes the power are typically fixed and not necessarily optimized for sonobioreactors. Therefore, most sonobioreactors are batch-type reactors with no control over ultrasound intensity inside the batch volume. Although the effect of ultrasound can be studied in this way, it fails to take into account the pressure distribution along the reactor volume. Finally, many studies rely only on the rated power of the ultrasound equipment, rather than the effective power transmitted into the reactor [18,19].

Here, we describe creating a novel reactor, in which ultrasound can be precisely controlled‬‬‬‬‬‬‬‬‬‬‬‬‬‬‬‬‬‬‬‬‬‬‬‬‬‬‬ to properly study its effect on biological systems.‬‬‬‬‬‬‬‬‬‬‬‬‬‬‬‬‬ An effective sonobioreactor is characterized by a powerful pressure response, whose resonance frequency has a symmetrical mode shape and an even intensity distribution. An adequate reactor is designed in such way that the structure’s vibrations have a good coupling to the fluid volume. The best coupling is achieved when the bending wave length matches the wave length in the fluid. The excitation points need to be located at the antinode of structural vibrations. For a specific reactor structure with a complex geometry, a multiphysics simulation model is necessary to define the vibration response and optimum excitation point that allows for a homogenous and strong pressure response in the fluid. The excitation is created by attaching a sonotrode directly to the vessel. A sonotrode is composed of a set of piezoelectric transducers and resonant metal rods used as an exciter of ultrasonic waves. However, to finalize the design of a sonobioreactor, experimental tuning and verification are required. For this purpose, we chose to test the novel equipment on two biotechnologically interesting microorganisms, the electroactive bacterium *Geobacter sulfurreducens*, and the lignocellulose-degrading fungus *Fusarium oxysporum*. Electroactive bacteria can generate electrical power through extracellular electron transfer, converting the chemical energy found in chemical bonds of organic compounds [20]. These bacterial systems have shown promise in various applications, including wastewater treatment, bioremediation or even as an alternative renewable energy source in remote areas [21–22]. Gram-negative *G*. *sulfurreducens* are known biofilm-forming electroactive bacteria [23]. In the absence of an electrode, fumarate and acetate are used as electron acceptor and donor, respectively, producing malate and succinate. *F*. *oxysporum* is a soil-dwelling filamentous fungus and one of the most important plant pathogens [24]. Its ability to produce several cellulases [25] and xylanase [26], as well as its ability to efficiently convert cellulose to ethanol in one step [27,28] makes it a promising microorganism for bioethanol production. In this study, the effect of ultrasound on the growth of these organisms and their metabolite production was investigated at different powers and resonating frequency of the proposed sonobioreactor.

## Material and methods

### Sonobioreactor design and construction

The design of the sonobioreactor comprised several steps. In the first step, a standard laboratory bottle was selected (250 mL, Duran GL 45 bottle) and a CAD geometry was converted to a finite element model. The eigenfrequencies were determined in a COMSOL simulation, and the corresponding eigenmodes of the pressure waves were visualized. The bottle had an eigenmode with a symmetric and centered pressure distribution within the bottle volume at a frequency of around 40 kHz. The accuracy of the model was then tested by tapping on the wall of a fluid-filled bottle and measuring the pressure spectrum inside the bottle. A strong response at the target frequency of around 40 kHz confirmed the accuracy of the model.

In the second step, to match the eigenfrequency of the bottle, the sonotrodes were designed to operate at around 40 kHz. This was done by modeling a sonotrode in COMSOL and optimizing the length of the masses. The designed sonotrode consisted of two piezoelectric discs (PZ 27, Ferroperm Piezoceramics) sandwiched between two stainless steel rod masses at the front and back (each steel mass was 24-mm long with an outer diameter of 10 mm). The masses were connected with a 7-mm long M4 screw. Each piezo disc had an outer diameter of 10 mm, an inner diameter of 4 mm, and a thickness of 2 mm.

In the third step, the sonotrode and the reactor bottle models were combined into a full sonobioreactor model to visualize the pressure distribution in the bottles. When the bottle was excited to vibrate at the resonance frequency, its structure was deformed and coupled to specific pressure modes. As a result, the model allowed us to estimate the frequency and mode shape in the bottle. The sonotrode was attached in a position that efficiently excited the mode shapes, with the pressure distributed evenly along the symmetry line of the bottle. The vibration in the bottle wall was transferred to the contained liquid as sound.

**Fig 1:**
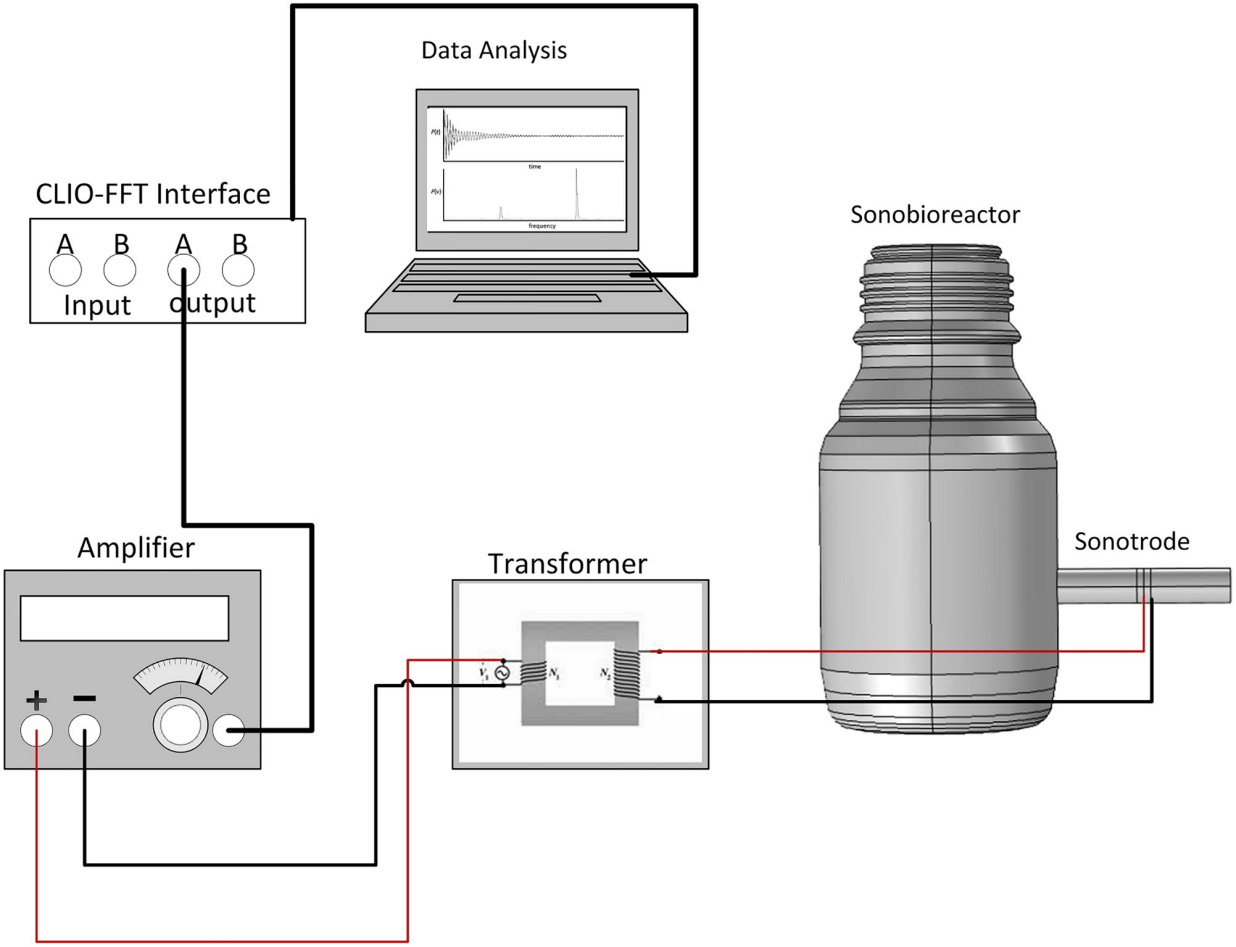
Schematic diagram of the experimental setup of the sonobioreactor.

The physical sonobioreactors were then constructed by gluing sonotrodes to the walls of standard laboratory bottles at the pre-determined position. The sonobioreactors were powered by an amplifier (MAC_2.2_ 2-channel power amplifier) with its voltage boosted by a transformer (gain of 8.9×). The input sound signal (pure sine) was generated using a PC-based software and hardware system (CLIO 12, Audiomatica), as illustrated in Fig 1.

Finally, the physical sonobioreactor was evaluated in terms of frequency responses and sound pressure. The CLIO system was used to measure the pressure response over time and frequency at a 192 kHz sampling rate. A signal conditioning amplifier (NEXUS 2692, Brüel & Kjaer) was used to connect a pressure sensor (2200V1, Dytran) and a miniature shock-type accelerometer (353M15, PCB) to the measuring system. The pressure transducer had a frequency range of 1 Hz to 100 kHz and higher. Both the CLIO output and input signals were controlled by an external CLIO-FFT interface.

### Modeling and quantification

The numerical model comprised a glass bottle structure and a sonotrode attached to the bottle wall. The vibration pattern and pressure distribution of the bottle were simulated using COMSOL Multiphysics software (version 5.3a). The simulation included three different modules: pressure acoustics, solid mechanics, and electrostatic. Each module was governed by its own equations as discussed hereafter.

In the simulation, the following assumptions were made: 1) some isotropic energy was lost due to piezoelectric effects or transmission of mechanical energy from the sonotrode; 2) the acoustic pressure distribution in the bottle was symmetric while damping of ultrasonic waves was negligible; and 3) the effect of cavitation bubbles generated in the bottle was also negligible.

Considering that the bottle material had a sufficiently high acoustic impedance, it was assumed to cause almost no absorption of incident ultrasonic waves. The displacement at the joint between the piezoelectric material and the stainless steel sonotrode was set to the same value. The default temperature was 293 K. The input information of the materials is summarized in S1 Table [29].

The pressure acoustics module was used to simulate ultrasound propagation in water. The acoustic wave equation was as follows [30, 31]:

$$\nabla \left(-\frac{1}{\rho }\nabla P+q\right)+\frac{\omega^{2}P}{\rho c^{2}}=0$$

Where ρ is the density of water (kg/m^3^), c is the speed of ultrasound propagation in water (m/s), P is the acoustic pressure (Pa), P = P_A_cos(ωt) P_A_ is the maximum acoustic pressure, ω is angular frequency (rad/s), t is time (s), and q is the dipole source (m/s^2^) which is optional. In our setup, there was no polarization (q = 0) for the longitudinal ultrasonic waves [36].

The boundary conditions set to couple the three modules followed COMSOL guidelines [32,33] and were based on previous simulation studies [8–9]. A structure-acoustic boundary was set to the interface between the structure of the bottle and water. Specifically, the vibrations of the bottle and the surrounding solution were coupled at the interface:

$$n\left(-\frac{1}{\rho_{s}}\nabla P+q\right)=\alpha_{n}$$

Where n is the normal unit vector, ρ_s_ is the density of the vibrating structure (kg/m^3^), and a_n_ is the normal acceleration of the fluid (m/s^2^).

The experimental part included testing the piezoelectric sonotrode before and after mounting it on a bottle. Experiments were conducted to determine impedance and transfer functions, resonance frequencies, pressure, and ultrasound intensity inside the bottle. The experimental procedures were carried out to define:

- The bottle frequency response by FFT measurements;
- The electrical power fed to the bottle;
- Power consumption by the liquid using the calorimetric method.

All of these aspects were monitored at various excitation frequencies and under controlled conditions of input power. Simulations were also performed in parallel. Finally, to verify the intensity distribution in the sonobioreactor the average pressure response was determined by pressure measurements at ten different positions.

### Microbes, media, and inoculation

*G*. *sulfurreducens* PCA (ATCC 51573, DSMZ 12127) obtained from Dr. Ashley Franks, La Trobe University, Bundoora, Australia, was used in all *Geobacter* studies. The *G*. *sulfurreducens* inoculum was grown in a slightly modified NBAF medium (pH 6.8), with acetate (20 mM) and fumarate (40 mM) as electron donor and acceptor, respectively, as described previously [34, 20]. A simpler medium, called “freshwater medium” (FWAF), containing 20 mM acetate and 40 mM fumarate was used in the sonobioreactor, as described previously [35]. The reactors were autoclaved, filled with FWAF in an anaerobic hood to ensure anaerobic conditions, and inoculated with 5% of NBAF inoculum.

*F*. *oxysporum* f. sp. lycopersici (CBS 123668) was purchased from Centraal bureau voor Schimmelcultures (CBS), Utrecht, The Netherlands, and kept on a PDA plate. The inoculum was prepared in yeast and malt extract medium (5 g/L and 20 g/L, respectively) and grown at 29 °C for 2 days. Then, a 6% v/v inoculum was used in a 250-mL flask containing 200 mL of 0.2 M sodium phosphate buffer, 0.3 g/L MgSO_4_·7H_2_O, 1 g/L KH_2_PO_4_, 10 g/L (NH_4_)_2_HPO_4_, and 1% w/v sucrose as carbon source.

The sonobioreactors were then placed in a temperature-controlled box (37 °C and 29 °C for bacteria and fungi, respectively) on a magnetic stir plate (200 rpm). Finally, the sonotrodes were connected to the amplifier and sonication was initiated at the desired intensity.

### Analytical methods

Biomass concentration of *G*. *sulfurreducens* was determined by measuring optical density at 600 nm. The concentrations of organic acids and sugars were determined using a Perkin-Elmer high-performance liquid chromatography (HPLC) system with a Series 200 refractive index detector, as previously described [36].

The biomass of *F*. *oxysporum* was determined by a combination of dry mass measurements up to day 3, as well as online biomass measurements using a Cell Growth Quantifier (CGQ; Aquila Biolabs). The pH of all samples was measured at the end of the experiment and compared with a control sample. Morphological changes due to ultrasonication were investigated by microscopic studies of platinum-coated ultrasound-treated and control samples by scanning electron microscopy (SEM) on a Magellan 400 instrument (Nanolab Technologies).

## Results and discussion

### COMSOL simulation

COMSOL simulations were conducted to estimate the sound pressure distribution and the high-pressure zones in the sonobioreactor volume. Numerically simulated results and verification of the physical sonobioreactor are described below.

### Simulation and verification of the sonotrodes’ response

**Fig 2:**
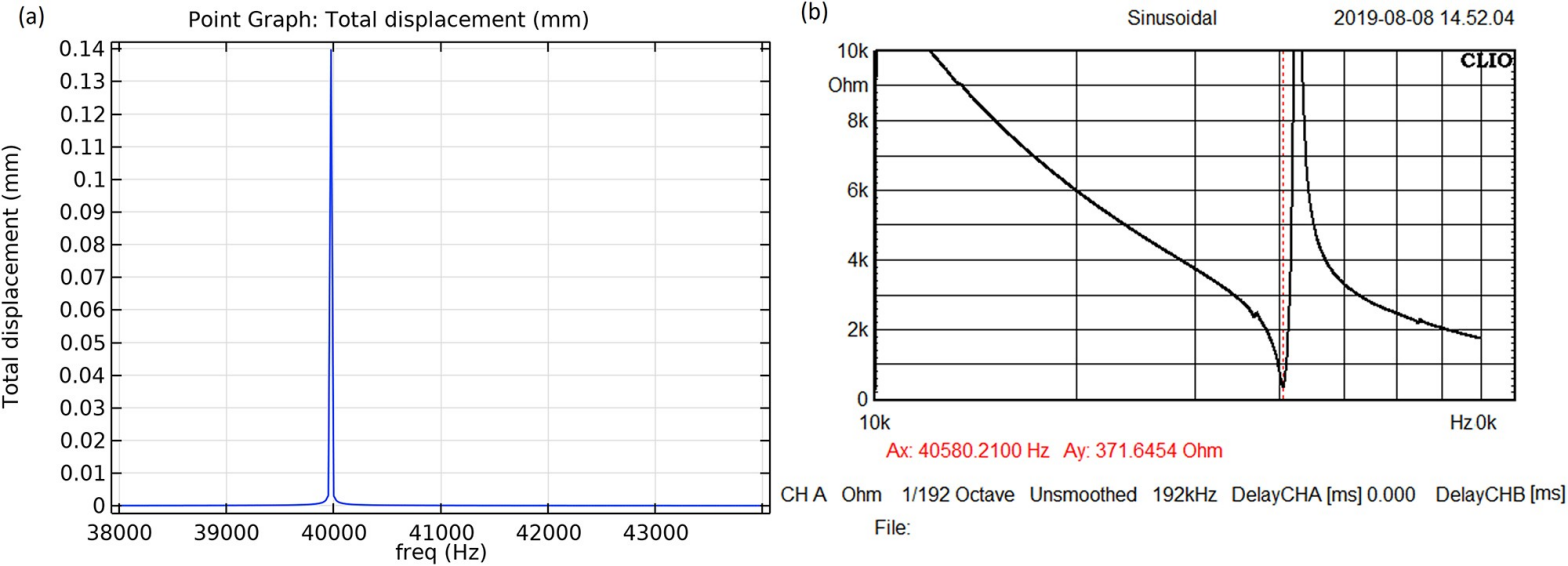
(a) Calculated displacement of sonotrodes from the model as a function of frequency. (b) Measured impedance of the physical sonotrodes in free field at a low input power.

The longitudinal displacement amplitude of the sonotrode model was calculated in the frequency range of 35–45 kHz. The length was adjusted to match the targeted resonance of the bioreactor at 40 kHz. Fig 2 shows the simulated displacement response and measured sonotrodes impedance as a function of frequency. The sonotrodes resonance in free field corresponded to the impedance minimum and occurred at a frequency of 40580 Hz. The minimum impedance of the sonotrode was 371 Ω.

### Simulated vibration pattern and pressure mode shapes

The vibration response of the sonobioreactor model was evaluated in the frequency range of 35–46 kHz. The expectation was to couple the bending wave of the reactor wall to the sonotrode’s standing wave pattern. Numerous resonances were detected in the frequency range of interest. Each resonance was linked to a specific mode shape of the system. Low output pressure or very high displacements in the reactor were not desirable.

**Fig 3:**
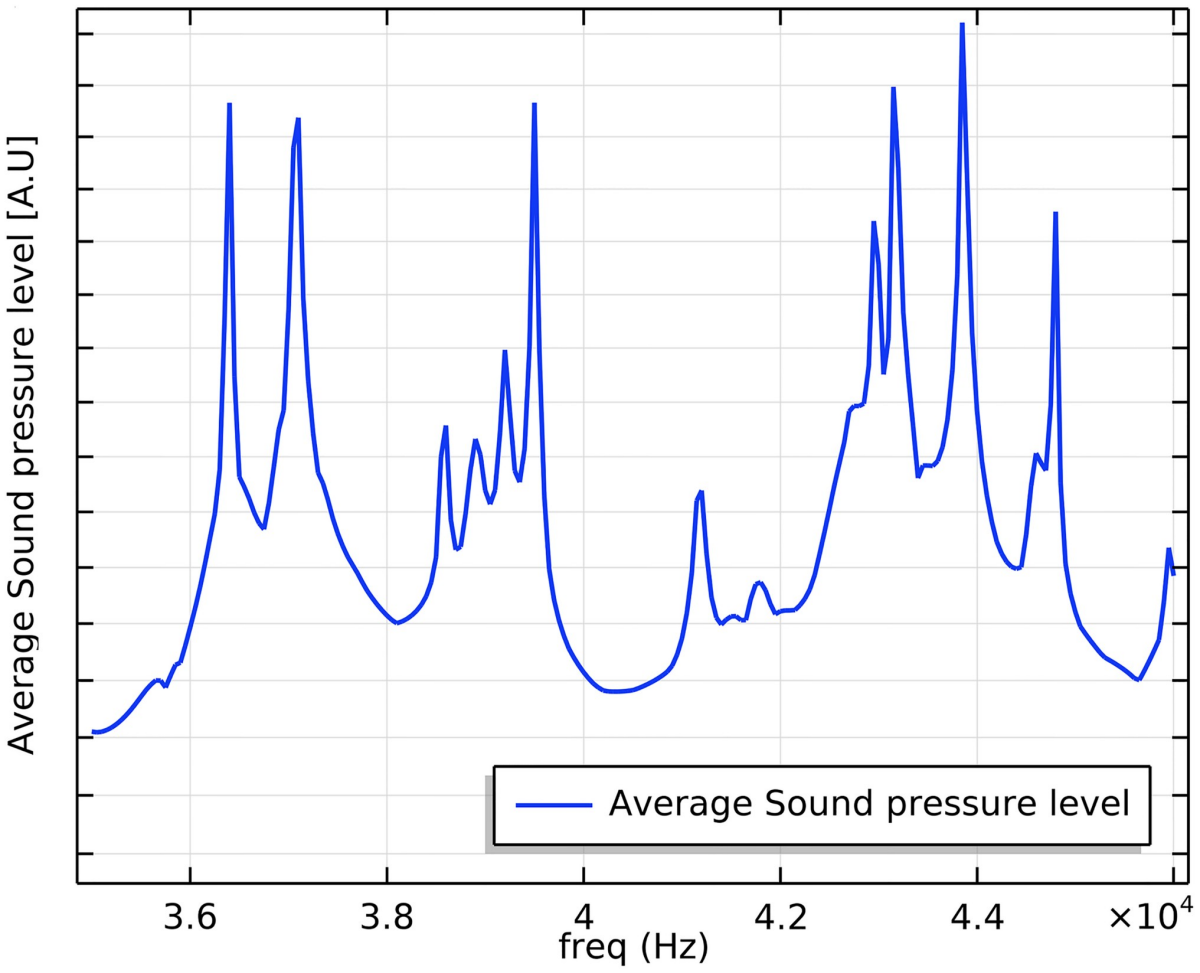
Average sound pressure distribution following excitation of the sonobioreactor with point force of 1 N.

**Fig 4:**
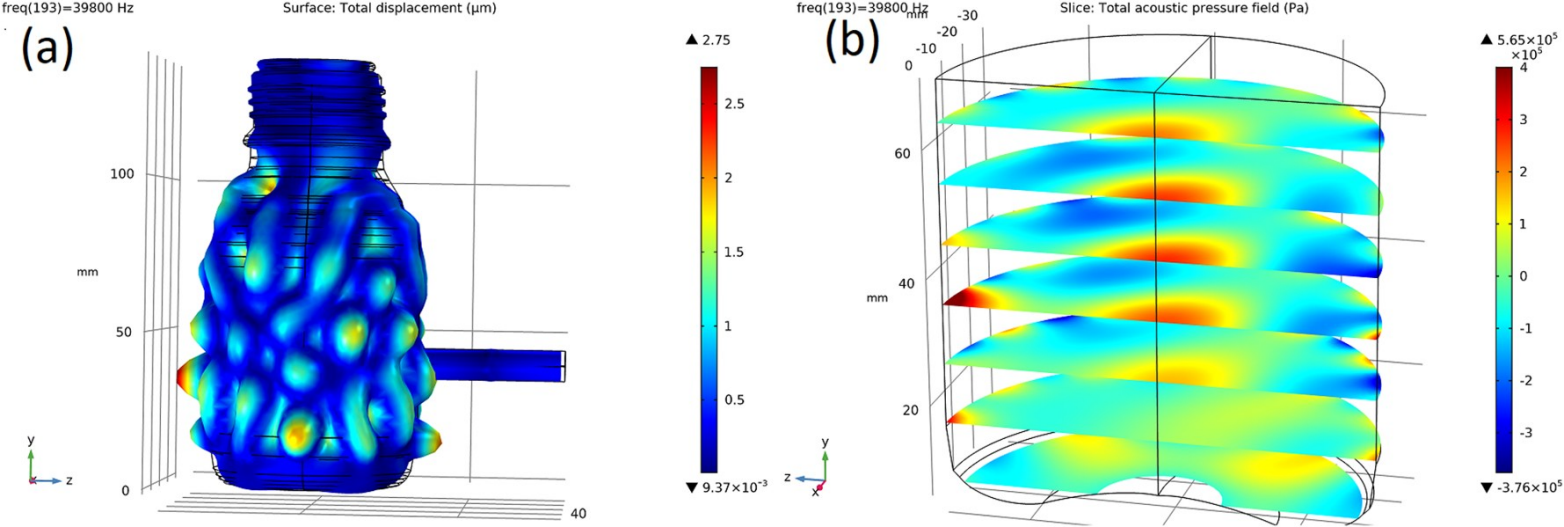
(a) Vibration pattern of the sonobioreactor and sonotrodes at 39.8 kHz. (b) Acoustic pressure distribution in the sonobioreactor at 39.8 kHz.

Fig 3 shows the averaged sound pressure level within the fluid volume of the sonobioreactor. The highest intensity was found at 39.8 kHz, where it was 10 times higher (+10 dB) than in the other modes. Fig 4 displays the displacement mode of the reactor at the highest intensity peak of 39.8 kHz. The reason that this frequency was chosen is that the model exhibited both a maximum pressure response at this frequency and an even pressure-distribution across the reactor volume.

The 3D-mode shapes of the whole setup provided a clear picture of the pressure distribution inside the simulated setup. To be useful, mode shapes should have the maximum pressure along the center of the reactor, whereas the walls of the reactor have lower pressure [37,38]. Mode shapes were evaluated for each peak in the specific frequency range to decide which one was useful.

The presence of multiple resonance frequencies is not surprising because of local phenomena guiding the reactor’s response. The resonance peak between 39 and 42 kHz was expected to provide the best coupling to the contained fluid. Amplitude peaks at different frequencies originated from interactions between other modes of the reactor. The simulated pressure response was determined by assuming that the water volume in the beaker behaved linearly. In practice, various aspects of the bottle, fluid, and sonotrode caused a nonlinear pressure response (such as cavitation bubble collapse), which was seen as a harmonic frequency response. Because of this nonlinear response, the peaks of the resulting pressure response tended to be less drastic (Fig 4).

#### Comparison between the model and the real system

**Fig 5:**
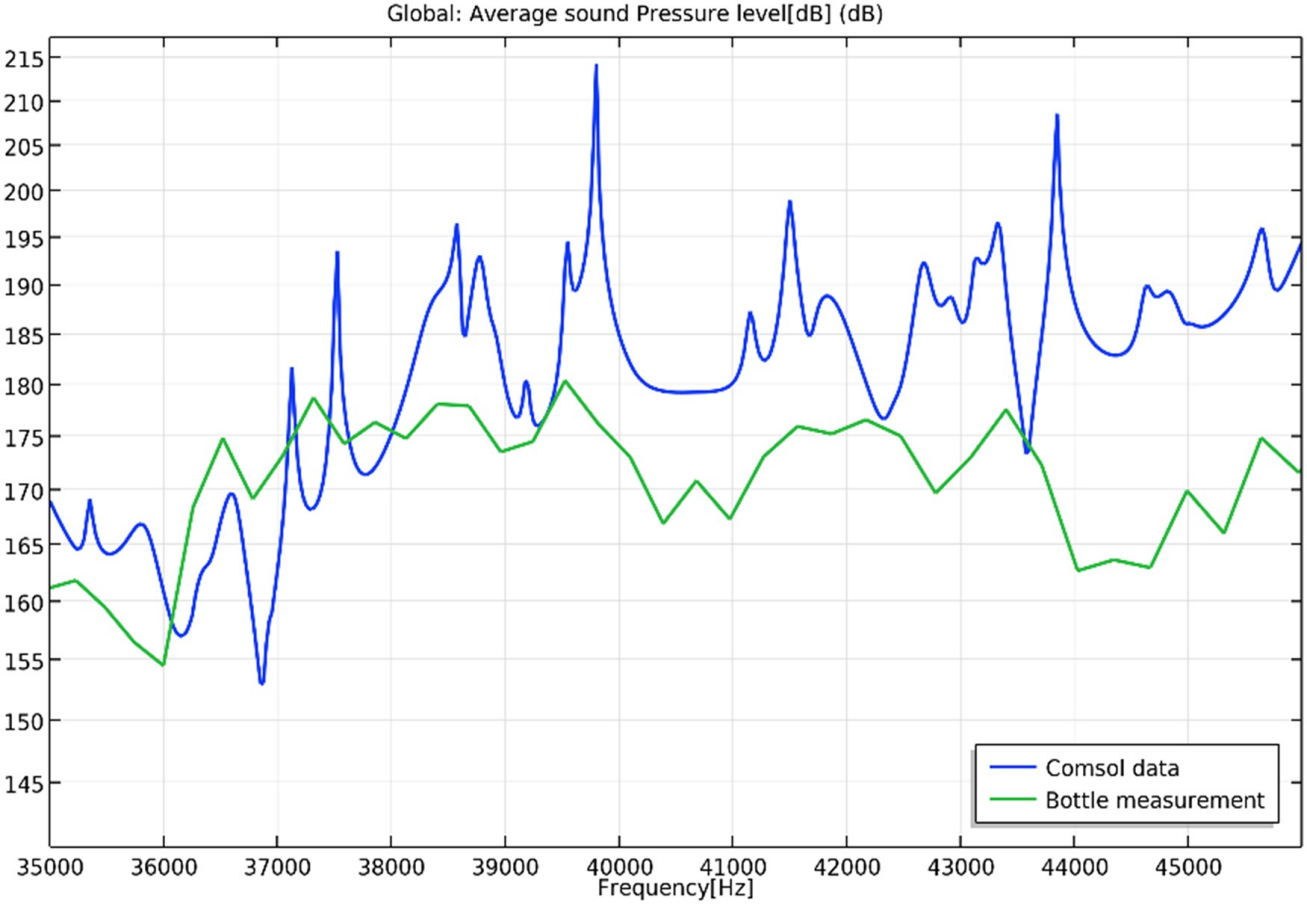
Simulated and measured frequency responses. The green line represents the average sound pressure measured by a pressure sensor at 10 different positions in the sonobioreactor filled with water (220 mL). The blue line represents the averaged simulated sound pressure level in the fluid volume.

In the experimental measurement, the peak response was observed at 39.5 kHz, which was close to the simulated resonance frequency (39.8 kHz). However, there were also noticeable differences at several frequencies (Fig 5). The smoothed characteristics of measurement response were due to a combination of nonlinear effects, low sampling resolution, and the averaging process.

The best match occurred near a well-defined impedance minimum, and coincided with the maximum pressure response in the reactor. The peak amplitude of the current signal’s frequency spectrum gave a good indication of when the maximum pressure response occurred. The current peak frequency represented an equivalent and simpler indicator than the impedance minimum. As a result, the frequency response could be determined without measuring pressure. At maximum response, current and voltage were slightly out of phase. The input electrical power corresponded to the product of the root mean square values of current and voltage, as well as the phase angle. With the system in operation, the "loss factor" (the energy lost during the transfer of sound waves) increased due to energy being transferred to the reactor, in the form of cavitation, energy transfer to the microorganisms, and frictional losses derived from the sonobioreactor’s structure.

### Calorimetric measurements

**Fig 6:**
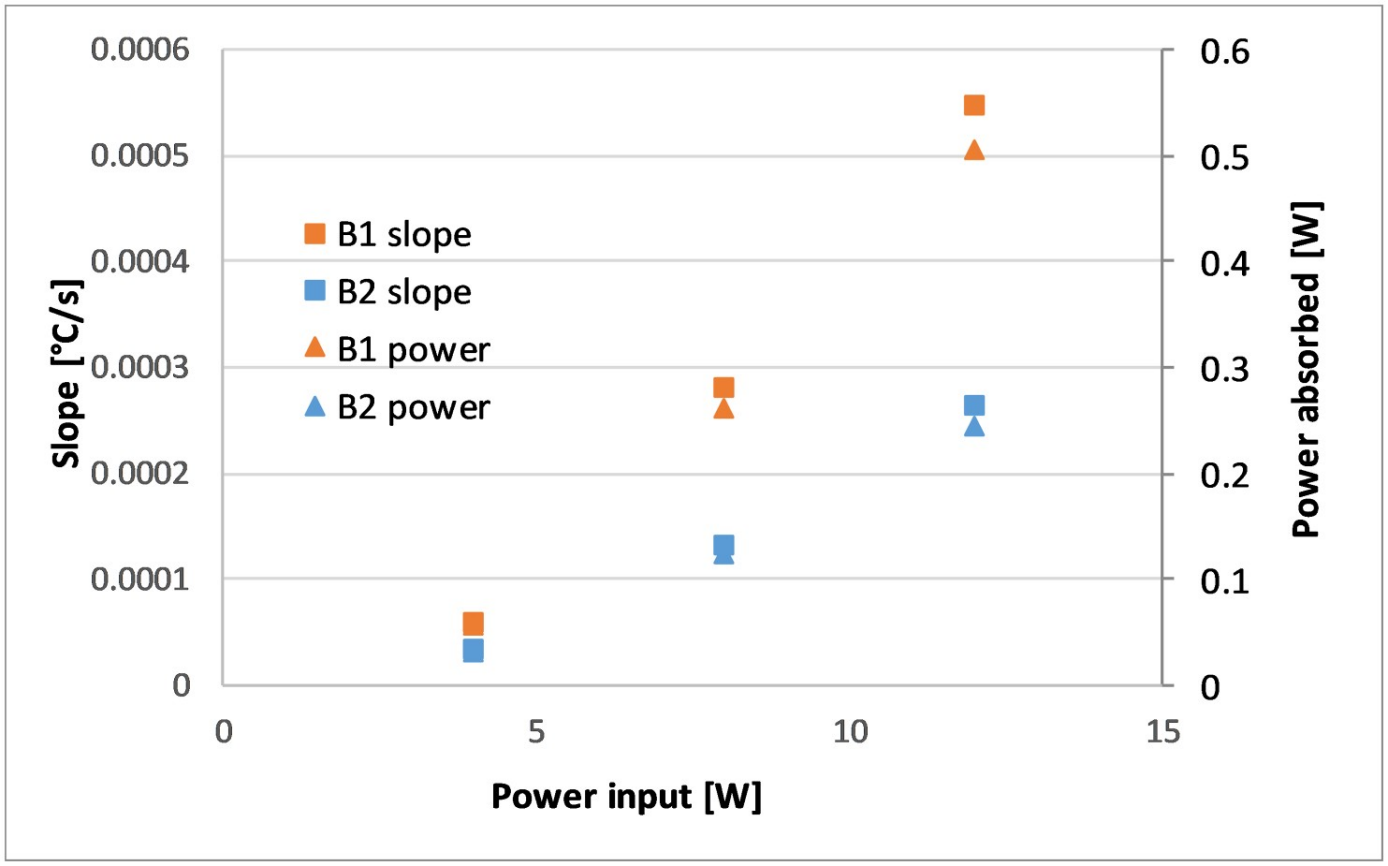
Calorimetric measurements of different bottles, showing the linear relationship between temperature increase and power level.

Calorimetric measurements were performed to further characterize the final energy input into the sonobioreactors. To this end, bottles filled with 220 mL deionized water were allowed to reach room temperature and then insulated well before temperature measurements were taken for several minutes. The increase in temperature was due to the energy absorbed by the water directly, which was related to the energy input into the sonobioreactor. The power input varied significantly depending on the resonance frequency of the specific bottle (Fig 6).

### Testing the sonobioreactors in biological systems

#### Effect of ultrasound on *G*. *sulfurreducens*

**Fig 7:**
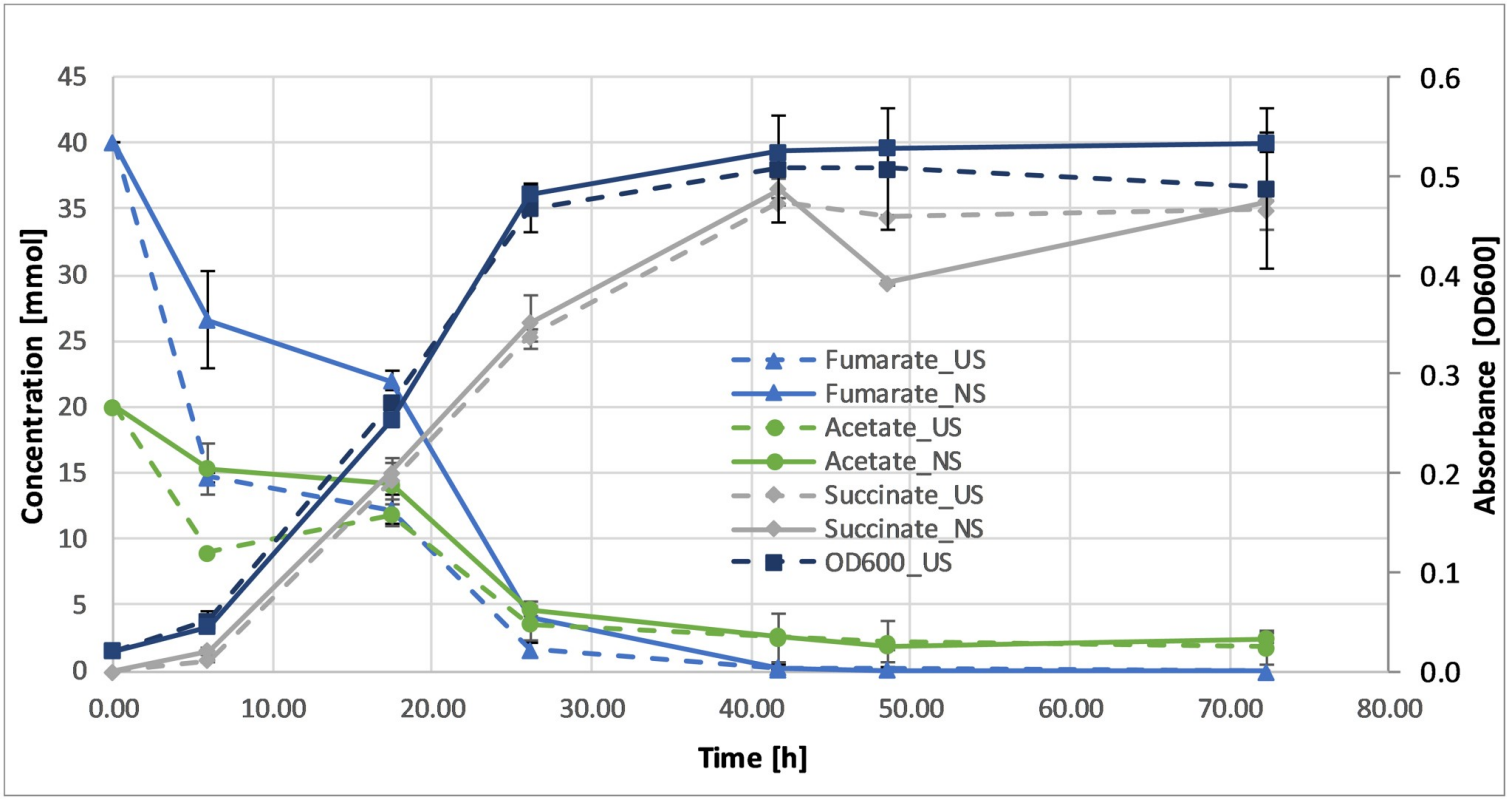
Metabolic profile and growth of *G*. *sulfurreducens* grown in the presence of ultrasound at 14 W power input (dashed lines) or in its absence (solid lines). Error bars represent standard deviations.

To determine the level at which ultrasound had an observable impact on the growth rate, cultures were grown at different power levels (4–14 W). The higher power levels caused the temperature in the bottles to increase; however, given that *G*. *sulfurreducens* is a thermotolerant species and does not lose viability when incubated at up to 40 °C, growth was not negatively affected [39]. Moreover, the growth rate of *G*. *sulfurreducens* was not significantly affected (Fig 7) even at higher power levels (12–14 W). The pH of the cultures did not change significantly during growth, with fumarate being the limiting factor for growth.

**Fig 8:**
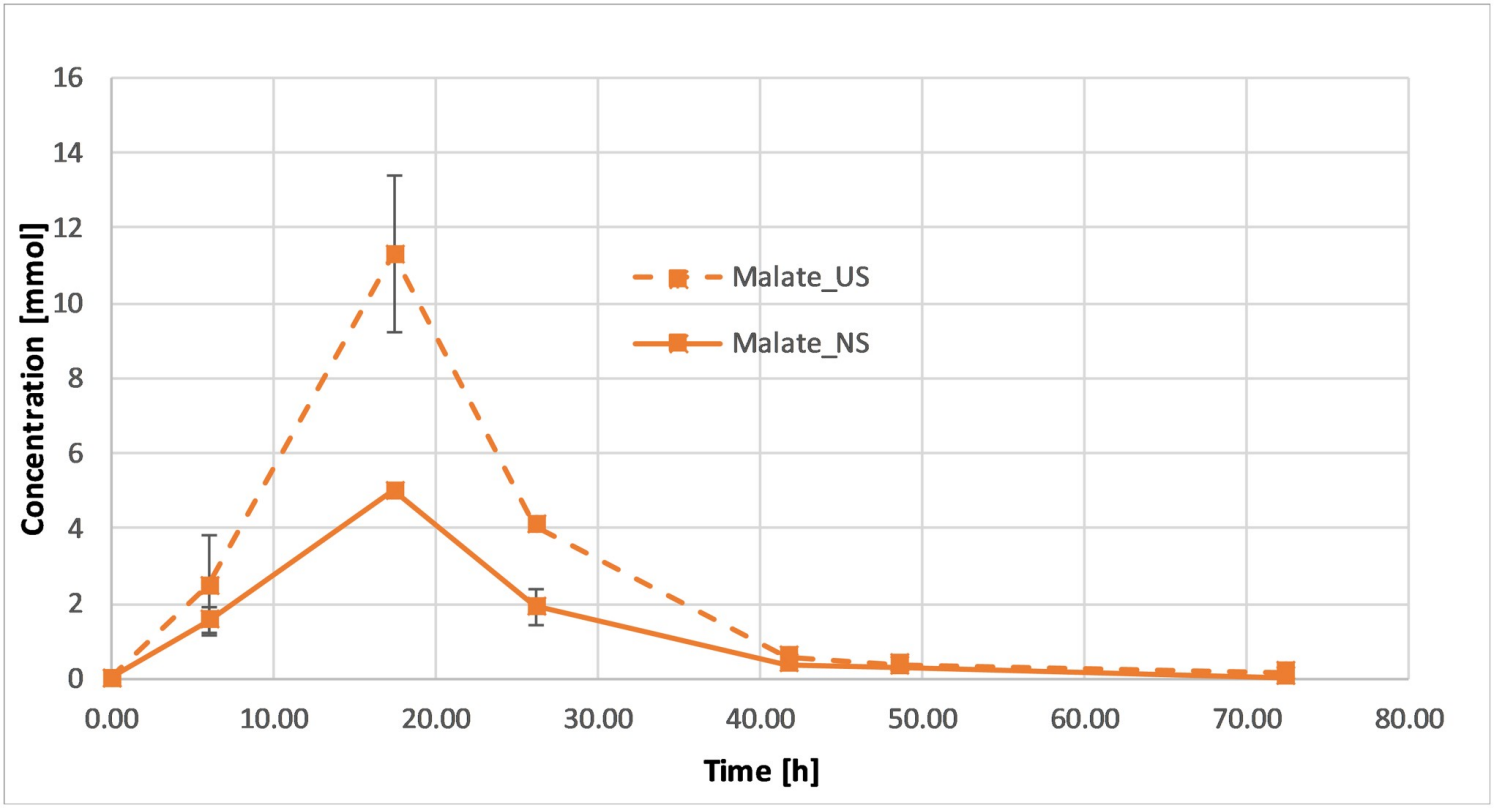
Malate profile of *G*. *sulfurreducens* grown in the presence of ultrasound at 14 W power input (dashed lines) or in its absence (solid lines). Error bars represent standard deviations.

During fermentation, malate is formed from fumarate and is then consumed, along with acetate, while succinate is produced. Fumarate and acetate consumption were similar in both the sonicated and non-sonicated fermentations, with the sonicated samples exhibiting a slightly increased rate during the first 5 h of fermentation. In contrast, succinate production was nearly identical for both types of samples (Fig 7). At high power inputs, however, a significantly higher malate concentration was obtained (11 mmol vs. 5 mmol) (Fig 8). This difference was not recorded at lower power levels. Currently, the reason for this change is not clear but could be ascribed to either a stress response related to a higher production of fumarase, or to an increase in membrane permeability, which enhances the excretion of malate. Several studies have shown an increase in permeability in a variety of cells resulting, for example, in higher fermentation efficiency or improved efficacy of antibacterial substances [40 –41].

#### Effect of ultrasound on *F*. *oxysporum*

**Fig 9:**
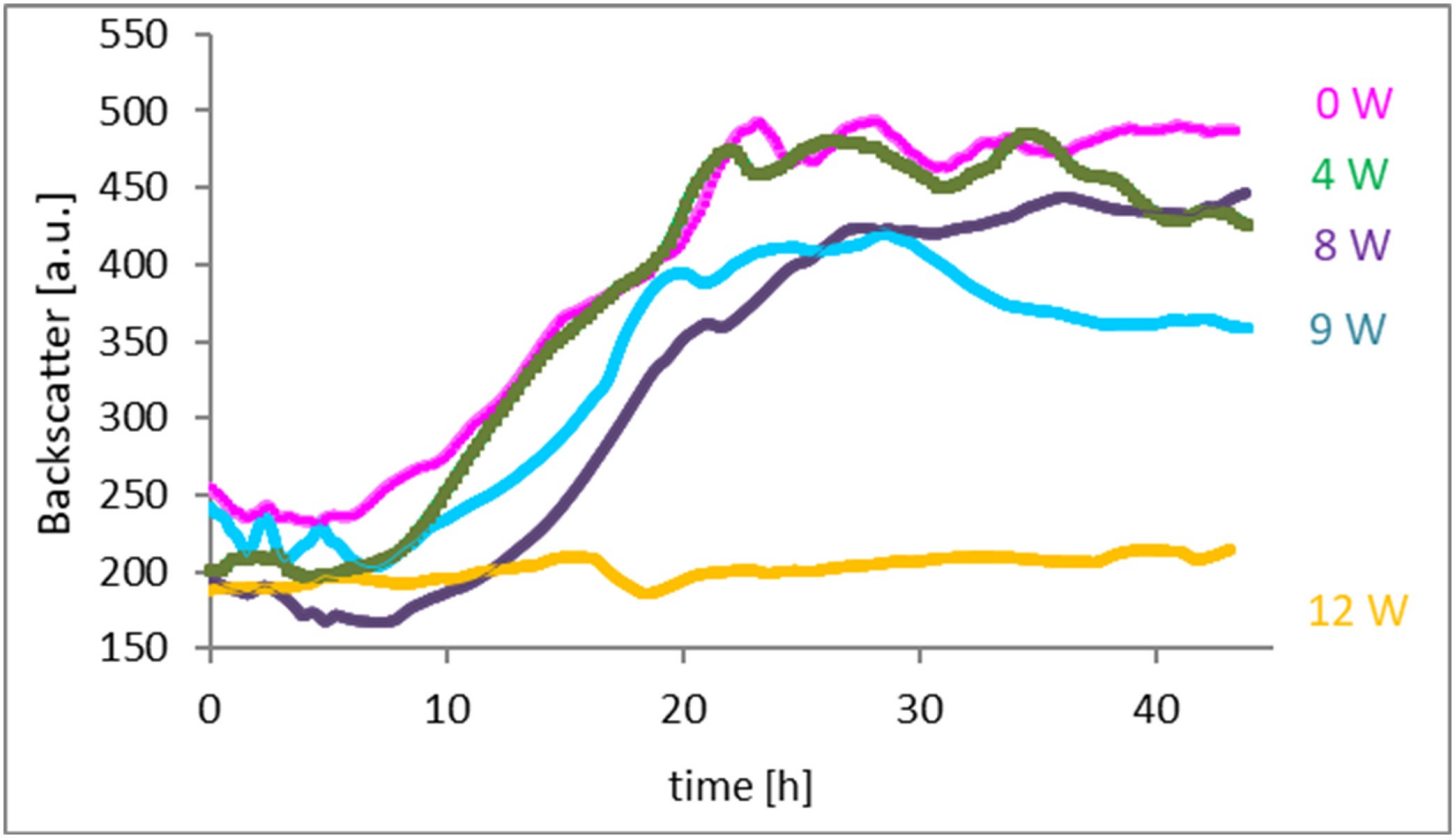
Fungal growth curve based on backscatter measurement by a CGQ.

**Fig 10:**
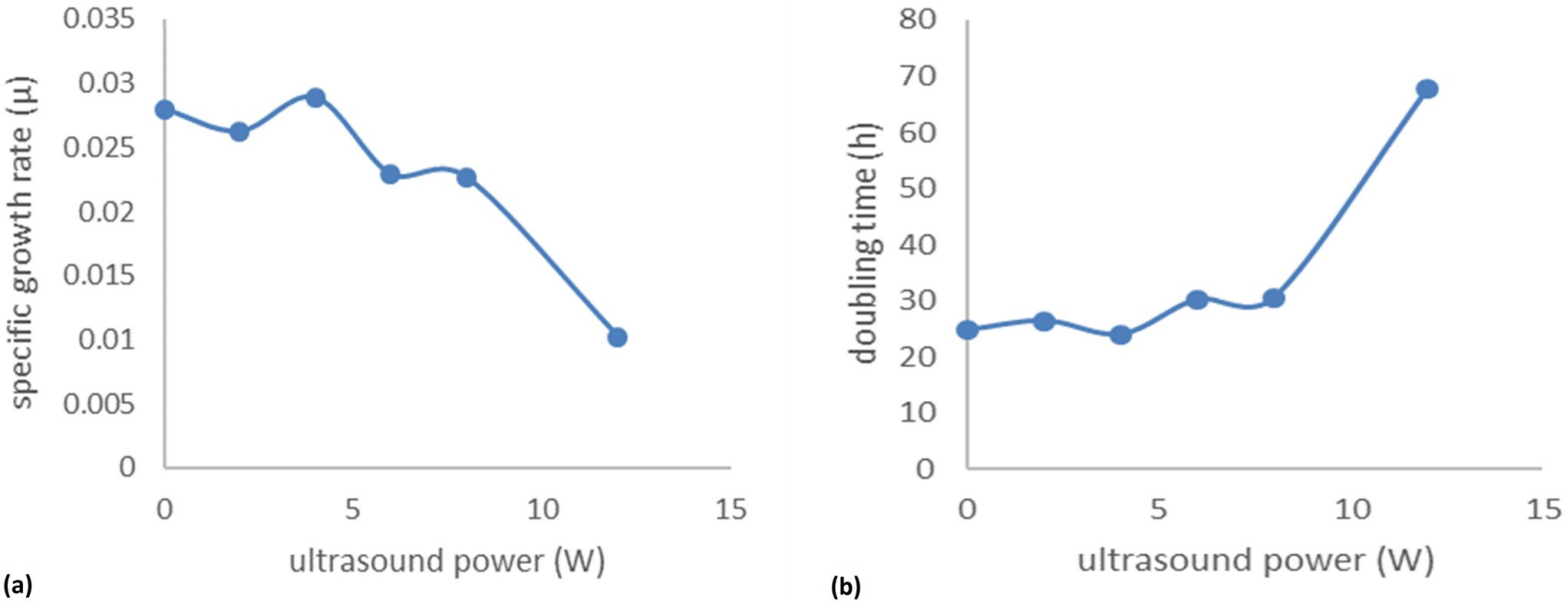
Effect of different ultrasound intensities on (a) the specific growth rate and (b) doubling time.

CGQ measurements at different input power levels (0–12 W) indicated that, unlike *G*. *sulfurreducens*, growth and morphology of *F*. *oxysporum* were more affected by ultrasonication. More specifically, input of 2–4 W did not affect fungal growth, but input energy ≥8 W had a negative effect on growth (Fig 9). This result was confirmed by measuring the dry cell weight (DCW) (S1 Fig), and calculating the specific growth rate and doubling time (Fig 10).

**Fig 11:**
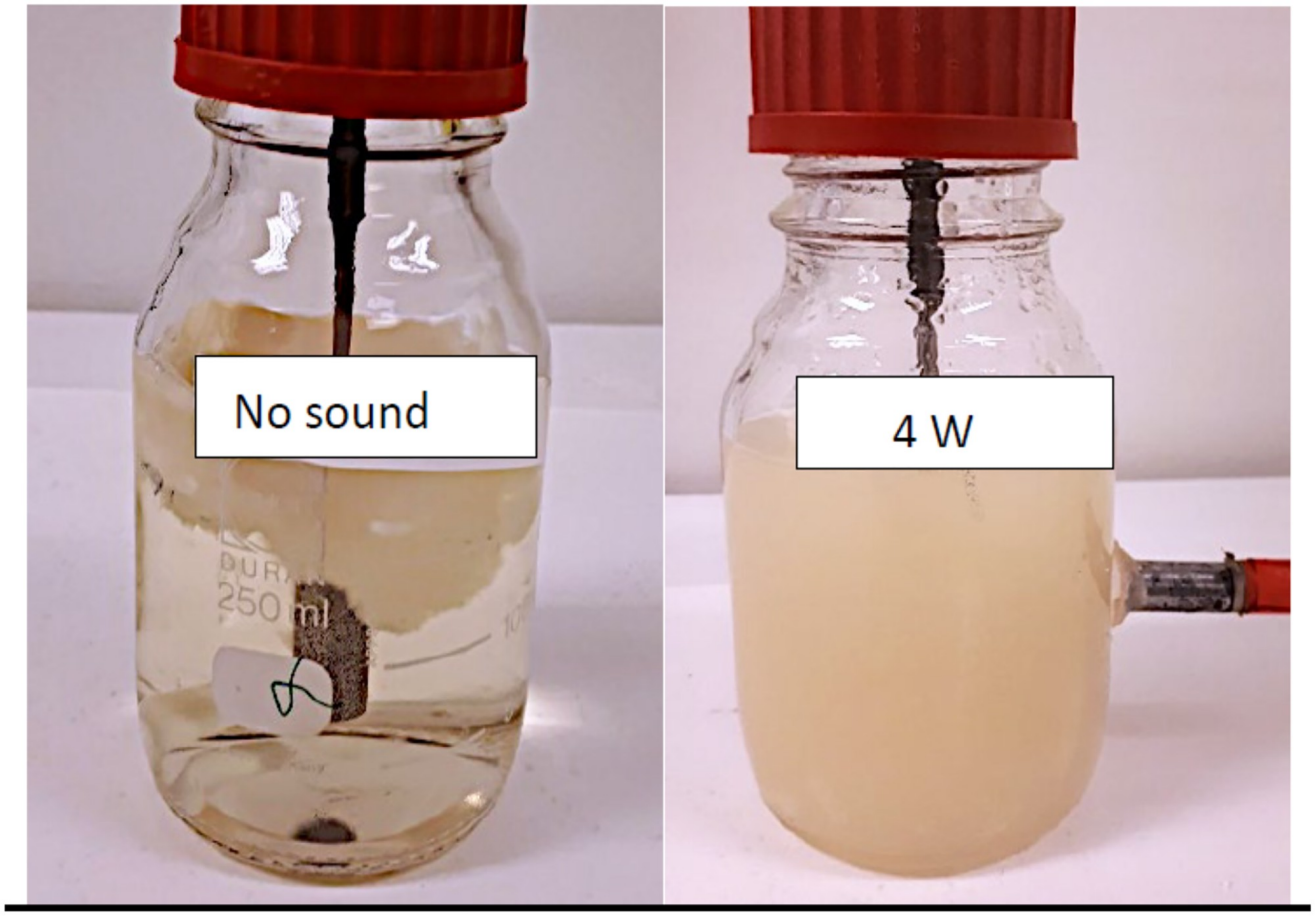
Morphological changes to *F*. *oxysporum* cultures resulting from increased ultrasound power.

**Fig 12:**
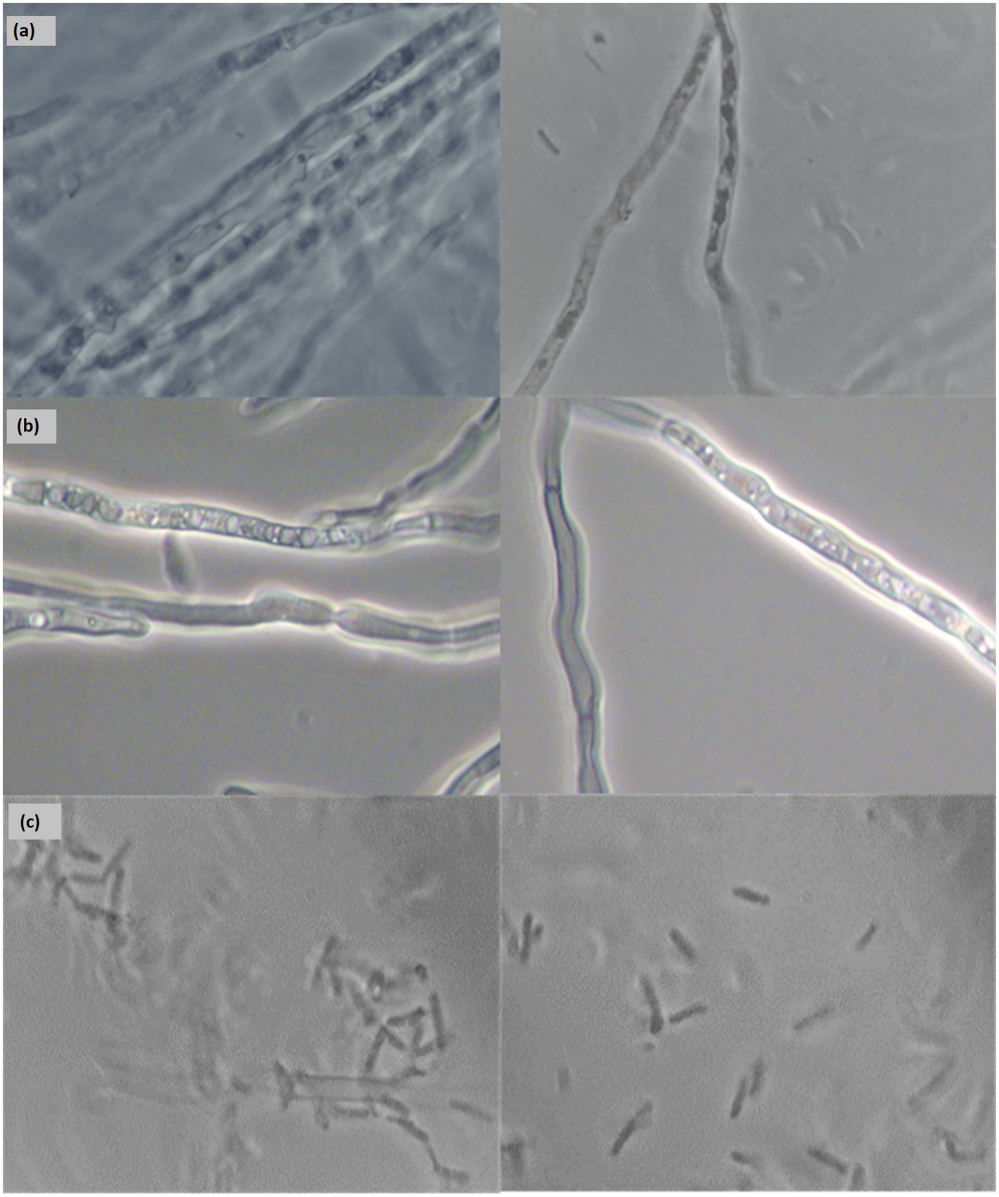
Micrographs of *F*. *oxysporum* at 100× magnification. (a) Control samples; (b) input power of 2 W; (c) input power of 4 W.

**Fig 13:**
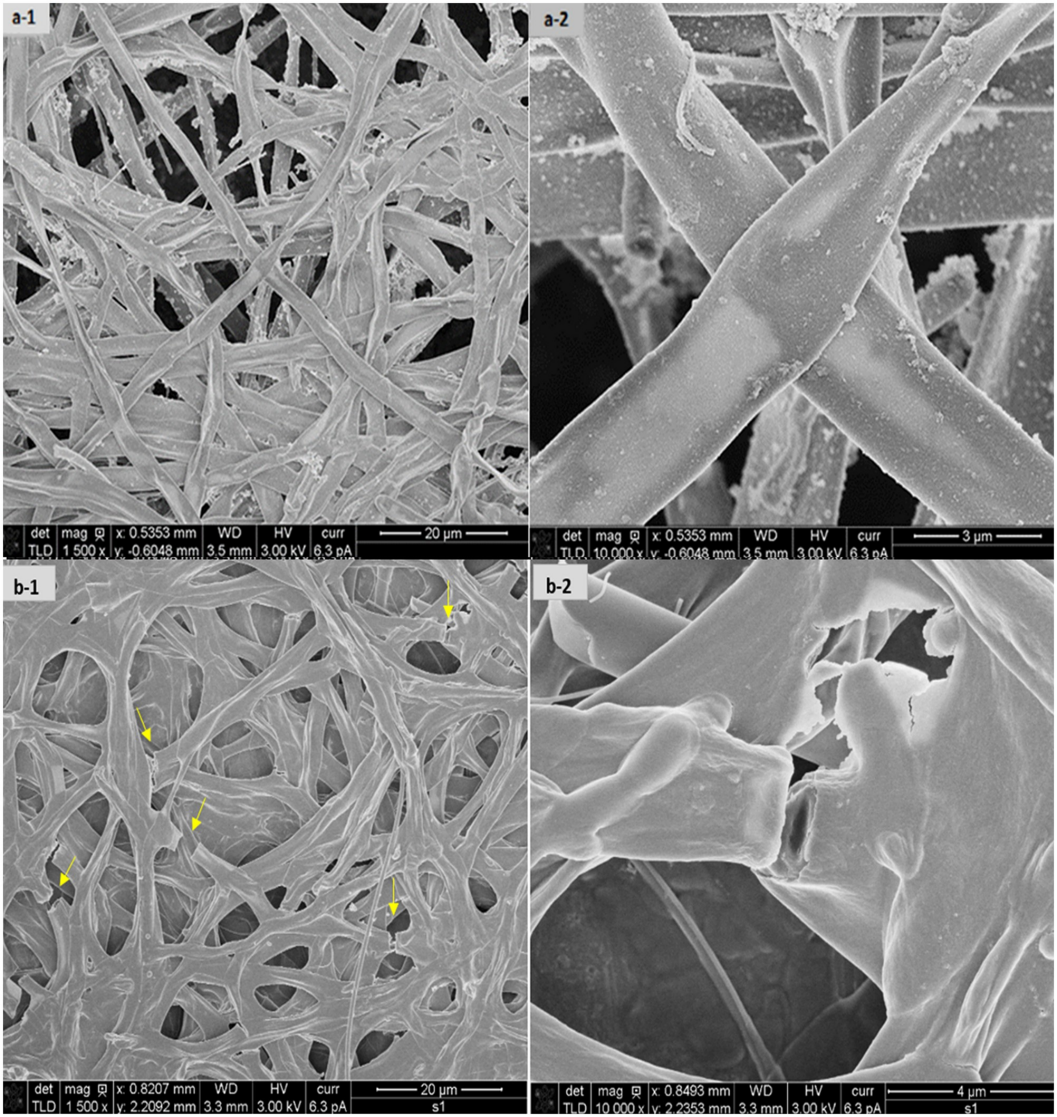
SEM images of (a) untreated and (b) ultrasound-treated (4 W) *F*. *oxysporum* biomass at different magnifications.

Besides a drop in total biomass, ultrasound changed also the morphology of the fungus. In control experiments, the fungus grew as a filamentous clump, accumulating around the sparger and on the liquid’s surface (Fig 11). A similar trend was observed at 2 W. However, at 4 W, the filaments broke into smaller pieces, resulting in a more homogeneous biomass akin to a bacterial or yeast culture (Fig 11). This observation was confirmed by observation of the cells in a microscope (Fig 12). Furthermore, SEM revealed the mycelia to be disrupted and the hyphae to be broken (Fig 13). These changes could be attributed to thermal and mechanical effects. More specifically, during ultrasound exposure, small bubbles may be formed and collapse as single bubbles or in a cloud, causing both an increase in temperature and shock waves that disintegrate the mycelium [42]. Moreover, rapid changes in the spatial gradient of acoustic intensity may create a radiation force that contributes to mycelial disintegration [43].

The observed morphological changes support the potential use of ultrasound as a morphology-modifying factor without decreasing biomass yield, which can be of interest in submerged fermentation where certain morphology types may interfere with bioreactor performance. Filamentous fungi can be grown either as individual filaments or as pellets [44]. In the latter case, the broth demonstrates Newtonian behavior, has lower viscosity, and allows better mass transfer that improves oxygenation [45]. However, large pellets can also limit internal mass transfer, decreasing product yield and promoting autolysis of cells in the central part of the pellet [44,46,47]. We believe that ultrasound may limit autolysis by decreasing the size of the pellets and improving nutrient and oxygen transfer. A correlation between fungal productivity and certain morphological states has been documented for citric acid [48] and penicillin [49]. For example, *Trcihoderma ressei* cellulase activity is controlled by the size of the mycelium and amount of biomass [50]; whereas pellets of *Aspergillus niger* possess increased glucoamylase activity and lower protease activity compared to the filamentous form [45]. Accordingly, besides optimizing medium composition, inoculum size, and agitation rate, controlled ultrasonication can serve to regulate cell morphology and production of desired products. In general, ultrasound waves create a stressful environment for microbial cells, which can respond by producing secondary metabolites of industrial importance. Previous studies have shown that low-intensity ultrasound could help increase biomass, because it produces steady cavitation and therefore forces microorganisms to actively produce the material required to repair damaged cells and continue proliferating [51]. However, higher ultrasound intensities lead to damage that cannot be repaired and may cause microbial death [51]. Here, increasing the input power to >8 W led to morphological changes and slower growth, which became even more profound at 12 W. Both thermal and mechanical effects can explain deformation and death. Online temperature curve analysis by CGQ at different power levels indicated an increase at 12 W (S2 Fig).

Ultrasonic waves can also be used to alter the physical properties of the environment and favor growth of the microorganism. For example, ultrasonication can efficiently decrease the size of hydrophobic substrates and increase their miscibility in the medium. This will make the substrate more accessible to the microorganism [52].

## Conclusion

In this study, a sonobioreactor was modeled, designed, and assembled. A sonotrode was optimized, using a numerical model, to match the resonance frequency of a standard laboratory bottle. This resulted in a sonobioreactor with a resonance frequency of approximately 40 kHz, which was confirmed by physical models. The pressure in the sonobioreactor was symmetric and centered along the bottle volume. Preliminary tests on two industrially important microorganisms showed that ultrasound intensity of the sonobioreactor could be controlled to alter microbial growth and morphology. Sonication resulted in increased malate production by *G*. *sulfurreducens*, but without affecting growth. In comparison, the morphology and growth of *F*. *oxysporum* were more sensitive to ultrasound intensity. Despite considerable morphological changes observed at 4 W input power, the growth was not adversely affected; however, at 12 W, growth was nearly halted. The designed sonobioreactor can be used in the future as a sonoreactor to modify enzymatic reactions or substrate hydrophobicity.

## Supporting information

S1 Table Material properties of the bottle and sonotrode. (TIF)

S1 Fig Effect of ultrasound input power on growth of *F*. *oxysporum* measured in terms of dry biomass. Error bars indicate the standard deviation.
(TIF)

S2 Fig Online temperature measurement by CGQ in the presence of different ultrasound input powers. (TIF)
