# Supplementary figures and images for: Numerical modeling and verification of a sonobioreactor and its application on two model microorganisms
Source: PLoS One. 2020 Mar 11;15(3):e0229738. doi: 10.1371/journal.pone.0229738 (PMC7065760; doi:10.1371/journal.pone.0229738)

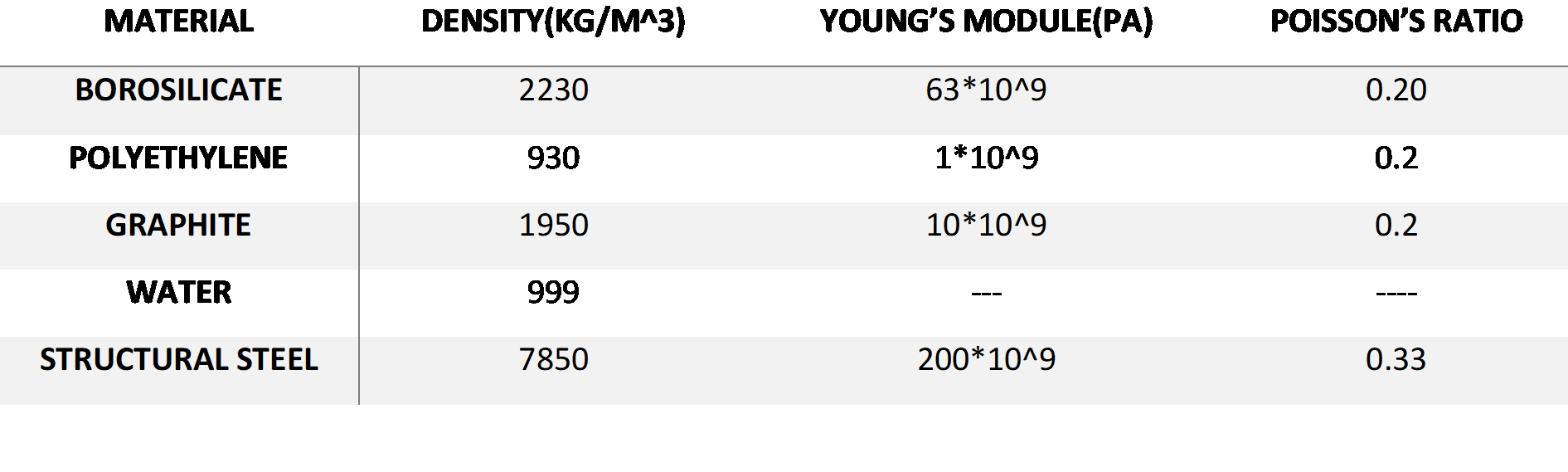

Supplement: S1 Table — (TIF) [file pone.0229738.s001.tif]

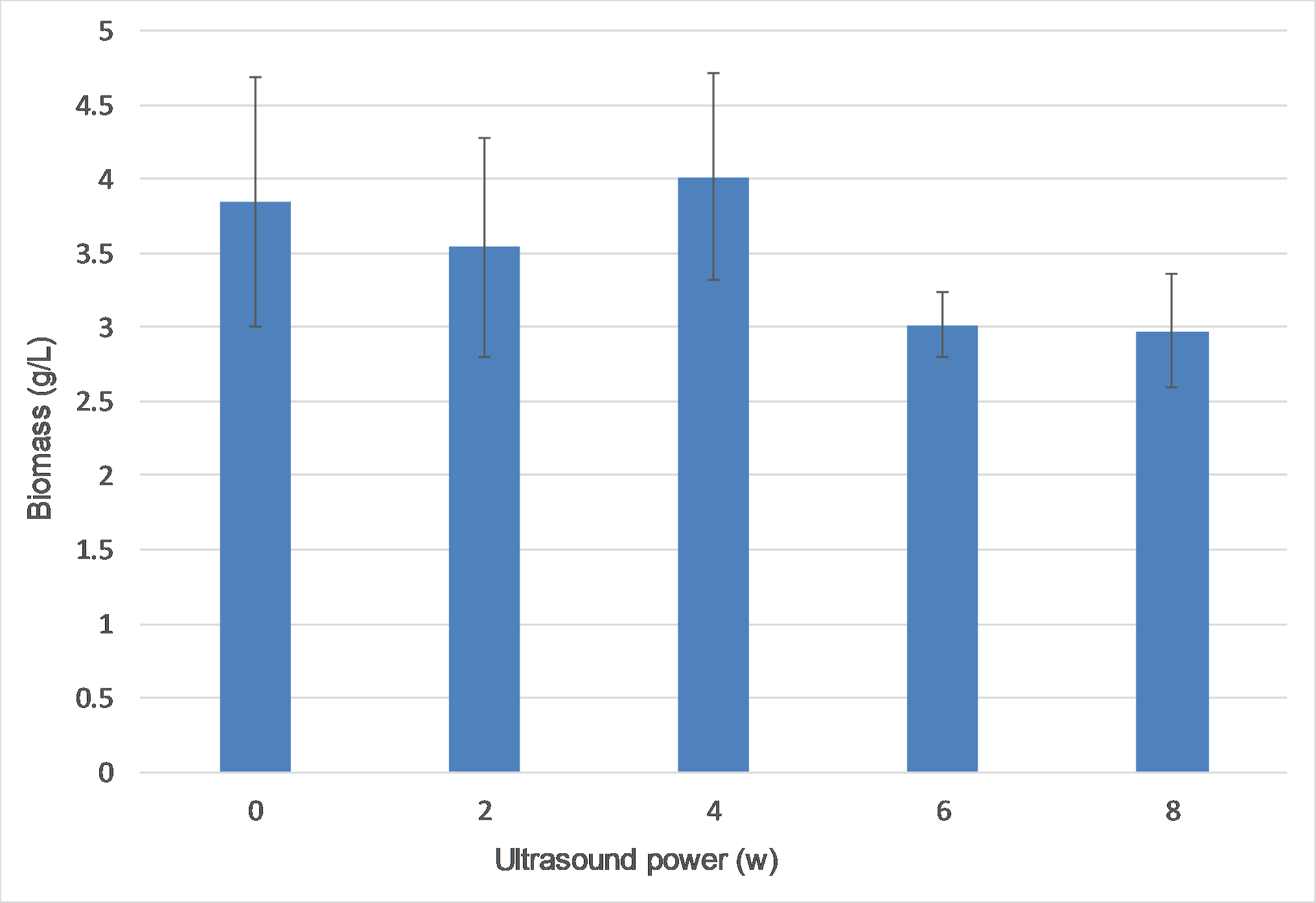

Supplement: S1 Fig — Error bars indicate the standard deviation. (TIF) [file pone.0229738.s002.tif]

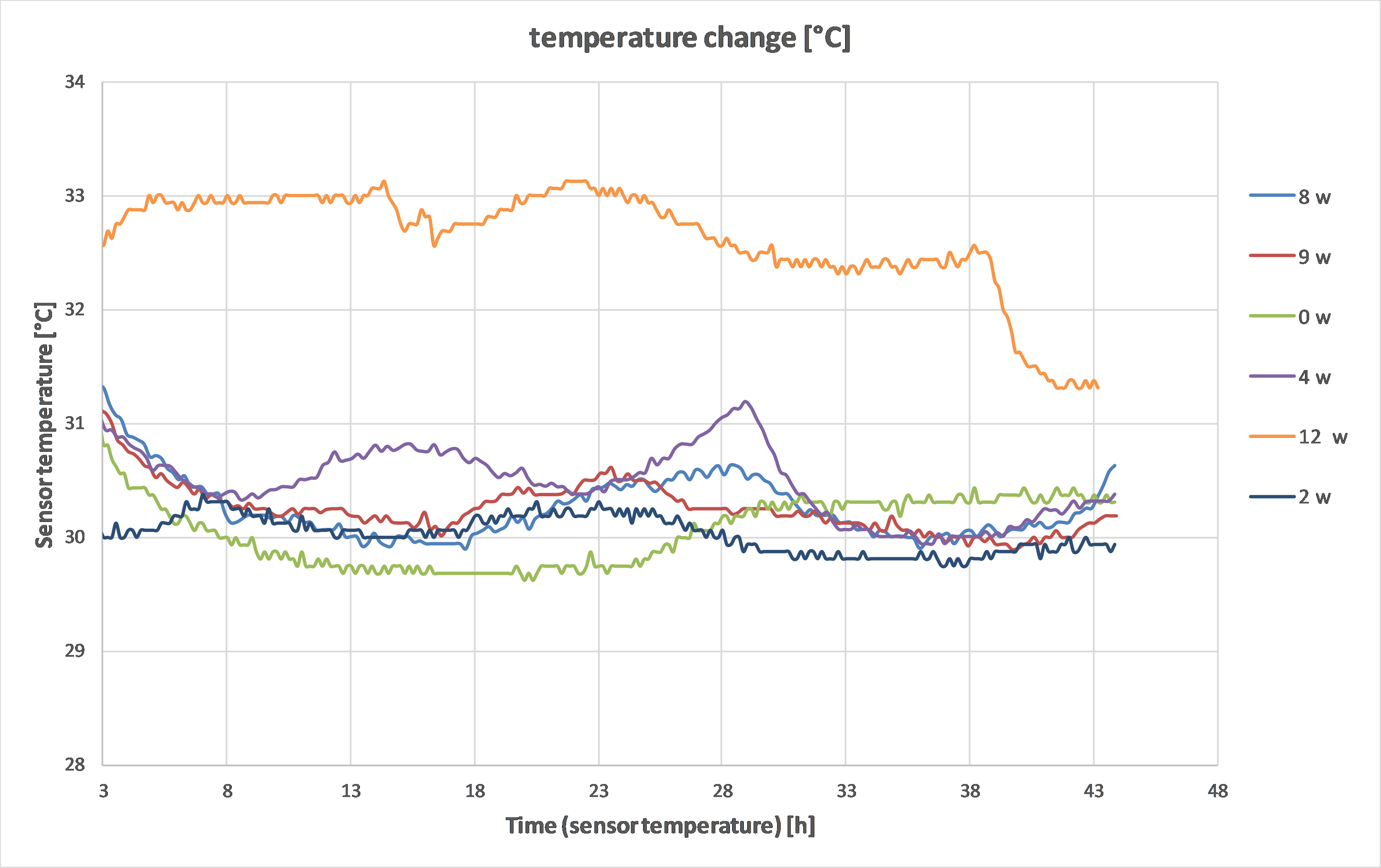

Supplement: S2 Fig — (TIF) [file pone.0229738.s003.tif]
